# Isolated tuberculous trochanteritis

**DOI:** 10.11604/pamj.2017.26.127.11719

**Published:** 2017-03-03

**Authors:** Zeineb Alaya, Walid Osman

**Affiliations:** 1 Department of Rheumatology, Farhat Hached Hospital, Faculty of Medicine of Sousse, Sousse, Tunisia; 2 Department of Orthopaedics, Sahloul Hospital, Faculty of Medicine of Sousse, Sousse, Tunisia

**Keywords:** Trochanteritis, tuberculosis, bone scintigraphy, bone biopsy

## Image in medicine

A 40-year-old patient was hospitalized for inflammatory pain in the superior-external surface of the right thigh evolving since 8 months, with no change in the general condition or fever. The pressure towards the right large trochanter was painful without local inflammatory signs. Biology did not show any inflammatory syndrome. The X-ray of the pelvis revealed an osteolytic lesion of the right great trochanter with thickening of the soft parts (A). Bone scintigraphy showed hyperfixation of the right great trochanter (B). The scanner of the basin revealed an osteolytic lesion of the right great trochanter containing multiple sequesters with cortical irregularity (C). A great-trochanter bone biopsy under scannographic control was made (D). The histological study showed an epithelioid and giganto-cellular granuloma with the presence of BK in the culture of the puncture fluid. The diagnosis of trochanteric tuberculosis was then made. Tuberculin intradermal reaction (IDR) was negative. The search for other tuberculous sites was negative. The patient was treated with anti-tuberculosis drugs during 12 months with good progression. Tuberculosis is a rare cause of trochanteritis. The peculiarity of our observation lies also in the absence of other tuberculous localizations.

**Figure 1:**
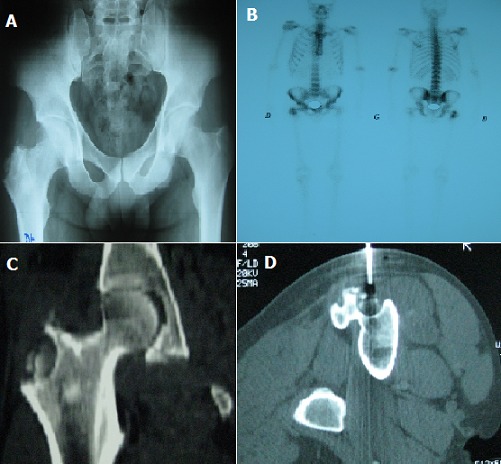
(A) X-ray of the pelvis: osteolytic lesion of the right great trochanter with thickening of the soft parts; (B) bone scintigraphy: hyperfixation of the right great trochanter; (C) scanner of the basin: osteolytic lesion of the right great trochanter containing multiple sequesters with cortical irregularity; (D) a great-trochanter bone biopsy under scannographic control

